# A comparison of standard occupational therapy versus early enhanced occupation-based therapy in a medical/surgical intensive care unit: study protocol for a single site feasibility trial (EFFORT-ICU)

**DOI:** 10.1186/s40814-021-00795-2

**Published:** 2021-02-18

**Authors:** Andrea Rapolthy-Beck, Jennifer Fleming, Merrill Turpin, Kellie Sosnowski, Simone Dullaway, Hayden White

**Affiliations:** 1 Logan Hospital, Brisbane, Queensland Australia; 2 School of Health and Rehabilitation Sciences, University of Queensland, Brisbane, Queensland Australia

**Keywords:** Critical care, Mechanical ventilation, Rehabilitation, Occupational therapy, Delirium, Cognition

## Abstract

**Background:**

Admissions to intensive care units (ICUs) are increasing due to an ageing population, and rising incidence of cardiac and respiratory disease. With advances in medical care, more patients are surviving an initial stay in critical care; however, they can experience ongoing health and cognitive limitations that may influence return to baseline function up to a year post-admission. Recent research has focused on the introduction of early rehabilitation within the ICU to reduce long-term physical and cognitive complications. The aim of this study is to explore the feasibility and impact of providing early enhanced occupation-based therapy, including cognitive stimulation and activities of daily living, to patients in intensive care.

**Methods:**

This study involves a single site randomised-controlled feasibility trial comparing standard occupational therapy care to an early enhanced occupation-based therapy. Thirty mechanically ventilated ICU patients will be recruited and randomly allocated to the intervention or control group. The primary outcome measure is the Functional Independence Measure (FIM), and secondary measures include the Modified Barthel Index (MBI), Montreal Cognitive Assessment (MoCA), grip strength, Hospital Anxiety and Depression Scale (HADS) and Short-Form 36 Health survey (SF-36). Measures will be collected by a blind assessor at discharge from intensive care, hospital discharge and a 90-day follow-up. Daily outcome measures including the Glasgow Coma Scale (GCS), Richmond Agitation and Sedation Scale (RASS) and Confusion Assessment Measure for intensive care units (CAM-ICU) will be taken prior to treatment. Participants in the intervention group will receive daily a maximum of up to 60-min sessions with an occupational therapist involving cognitive and functional activities such as self-care and grooming. At the follow-up, intervention group participants will be interviewed to gain user perspectives of the intervention. Feasibility data including recruitment and retention rates will be summarised descriptively. Parametric tests will compare outcomes between groups. Interview data will be thematically analysed.

**Discussion:**

This trial will provide information about the feasibility of investigating how occupational therapy interventions in ICU influence longer term outcomes. It seeks to inform the design of a phase III multicentre trial of occupational therapy in critical care general medical intensive care units.

**Trial registration:**

Australia New Zealand Clinical Trials Registry (ANZCTR): ACTRN12618000374268; prospectively registered on 13 March 2018/https://www.anzctr.org.au

Trial funding: Metro South Health Research Support Scheme Postgraduate Scholarship

**Supplementary Information:**

The online version contains supplementary material available at 10.1186/s40814-021-00795-2.

## Background

Admissions to intensive care units within Australian hospitals have increased over the last 10 years with approximately 161,000 patients requiring a stay in an intensive care unit between 2017 and 2018 [1]. The long-term burden of care and admissions requirements may also change in accordance with unpredictable disease presentations and an increasing requirement of health care service provision.

Patients admitted to an intensive care unit may experience a lack of control, reduction in sensory stimulation and reduced engagement in meaningful activities [2], in addition to being subjected to intrusive interventions. Subsequently, patients may develop a sequelae of cognitive and physical symptoms known as post-intensive care syndrome (PICS), often accompanied by reduced long term participation outcomes [3, 4]. Longitudinal studies on critical illness survival show that 30–80% of patients will acquire PICS, characterised as a collection of complications including persistent cognitive dysfunction, acquired weakness and post-traumatic stress disorder [5, 6]. A substantial proportion of patients who survive their initial critical care stay report significant decreases in quality of life at 3–6 months post admission [4, 7] in addition to increased mortality and associated economic costs [8–10], especially in the presence of acute respiratory distress syndrome (ARDS) [11].

Early rehabilitation within critical care settings is now considered effective and feasible when carried out within a multidisciplinary approach [12–16] following successful pioneering studies first conducted in 2009 [17, 18]. Yet while physical rehabilitation and early cognitive stimulation addressing delirium management have been shown to be effective [12], to date, there remains limited evidence regarding the impact of early task-specific training carried out by occupational therapists directed towards cognitive and functional engagement within the intensive care setting [19]. Altering the approach to rehabilitation through participation in meaningful cognitive and functional tasks such as activities of daily living rehabilitation may lead to further long-term benefits. Critical care practice has not traditionally included occupational therapy engagement in functional tasks or early self-care rehabilitation; thus, there is a growing need for early effective interventions addressing cognitive, physical and psychological functioning to optimise long-term functioning [20]. While occupational therapy service delivery and role in intensive care units may vary between critical care settings [19, 21], its relevance and impact remains supported by a recent systematic review [22] and continues to be explored with respect to scope of practice and benefits [23]. Costigan et al. [19] in their scoping review note that occupational therapy interventions in the intensive care remain dominated by physical rehabilitation (splinting, positioning) and less by practice of activities of daily living. This feasibility trial will consider splinting and positioning as standard usual care and introduce occupation-based therapy (using activities of daily living practice) as the intervention for comparison.

A recent study by Alvarez et al. [24] demonstrated the effectiveness of occupational therapy for managing delirium in non-mechanically ventilated patients and provided a basis for the introduction of occupational therapists as core multidisciplinary team members to address intensive care unit-related disorders during recovery. However, there is an ongoing need for research into the feasibility and effectiveness of graded occupation-based activities within the intensive care unit for patients who are mechanically ventilated. Howell [2] provided an approach for delivering self-care tasks to patients in intensive care. Howell proposed that, through participation in structured and graded purposeful activities and occupations, the reticular activating system which controls the brain’s ability to interpret and respond to stimuli is challenged such that a state of sensory overload or sensory deprivation is avoided [2]. Appropriately challenging activities lead to positive cognitive and sensory stimulation, which affects long-term participation by improving physical strength and functional ability [2]. Participation in purposeful activities and task-specific training is supported by multiple publications [25, 26], including three reviews [27–29], that address various aspects of task-specific training delivery and encourage the use of structured and individualised activities of daily living as a therapeutic medium. The majority of supportive literature relates to the diagnosis of stroke, with a Cochrane review [30], finding that patients who participate in occupational therapy interventions are less likely to deteriorate and more likely to gain higher levels of independence in activities of daily living. Support for the introduction of task-specific training in distinct populations such as Alzheimer’s disease [25] and total hip replacement [26] leads to varying results; however, the paucity of literature does not reflect usual clinical practice. Meaningful activities improve cortical reorganisation, which in addition to regular practice and sufficient intensity, impact significantly on recovery within the domain of self-care [31]. The literature may focus predominantly on the stroke population [32], yet these concepts may be transferable to patients who endure an intensive care admission, due to higher exposure to mechanical ventilation, ongoing sedation and subsequent prolonged bed rest leading to weakness. Task-specific training can therefore be defined as “training which utilises, as its principal therapeutic medium, ordinary everyday activities which are intrinsically and/or extrinsically meaningful to the patient” (pg. 181) [33], and often includes activities of daily living or occupation-based therapy.

Engagement in functional rehabilitation activities within the critical care setting needs to be explored further. While the critical care patient cohort is often unwell, integrating rehabilitation and participation within the limits of their medical stability has beneficial effects from a user perspective to offset the feelings of anxiety, loneliness and fear when mechanically ventilated [34, 35]. There is limited evidence exploring the patient perspective on participating in structured rehabilitation however exploration of factors such as diary use [36], humanisation of nursing environments [37] and relatives’ experiences of intensive care admissions [38] support a qualitative approach towards understanding factors that may influence participation in occupation-based therapy.

### Aims and objectives

The aim of this study is to explore the feasibility and impact of providing early enhanced occupation-based therapy, including cognitive stimulation and activities of daily living, to patients in intensive care. The intervention will focus on cognitive stimulation and engagement in functional activities in mechanically ventilated patients within an intensive care setting. This will be explored through a pilot feasibility trial comparing standard care to daily early enhanced occupation-based therapy. It is hypothesised that critically ill mechanically ventilated patients who participate in the intervention will demonstrate increases in functional ability, cognition and participation compared to those patients who receive standard care. Feasibility aspects of the intervention to be investigated include whether occupational therapy can be provided consistently to a partially sedated population, as well as within a medical setting where environment and medical equipment may limit interventions.

The study is divided into two parts: Part 1 aims to investigate the feasibility of early enhanced occupation-based therapy in a medical/surgical intensive care unit. Part 2 aims to explore the perceptions of participants and their significant others in relation to the therapy experience. Both parts of the study are carried out concurrently.

Specifically, the objectives of the early enhanced occupation-based therapy (the intervention) are:

Part 1:

1. To evaluate the utility of a selection of functional, cognitive, physical and quality of life outcome measures in their ability to capture the effect of the intervention on participants and their long-term outcomes.
2. To identify recruitment and consent rates from eligible participants
3. To evaluate retention rates in response to follow up procedures (attendance at interviews)
4. To test the intervention in terms of therapist compliance, ability to provide consistent intervention (fidelity), differentiation from current usual practice and amount of staff skill/experience required to carry out the intervention
5. To explore the effect of the intervention on occupational performance at intensive care and hospital discharge, and to evaluate the effect on cognitive, functional and mood factors on discharge from the intensive care unit and hospital, and at 90 days post-randomisation.

Part 2:

1. To qualitatively investigate the perception of the occupational therapy intervention with the experimental group participants and their significant others, exploring factors such as experience of participation, satisfaction with treatment content and perceived benefit of participation.

## Methods

### Design

**Fig. 1:**
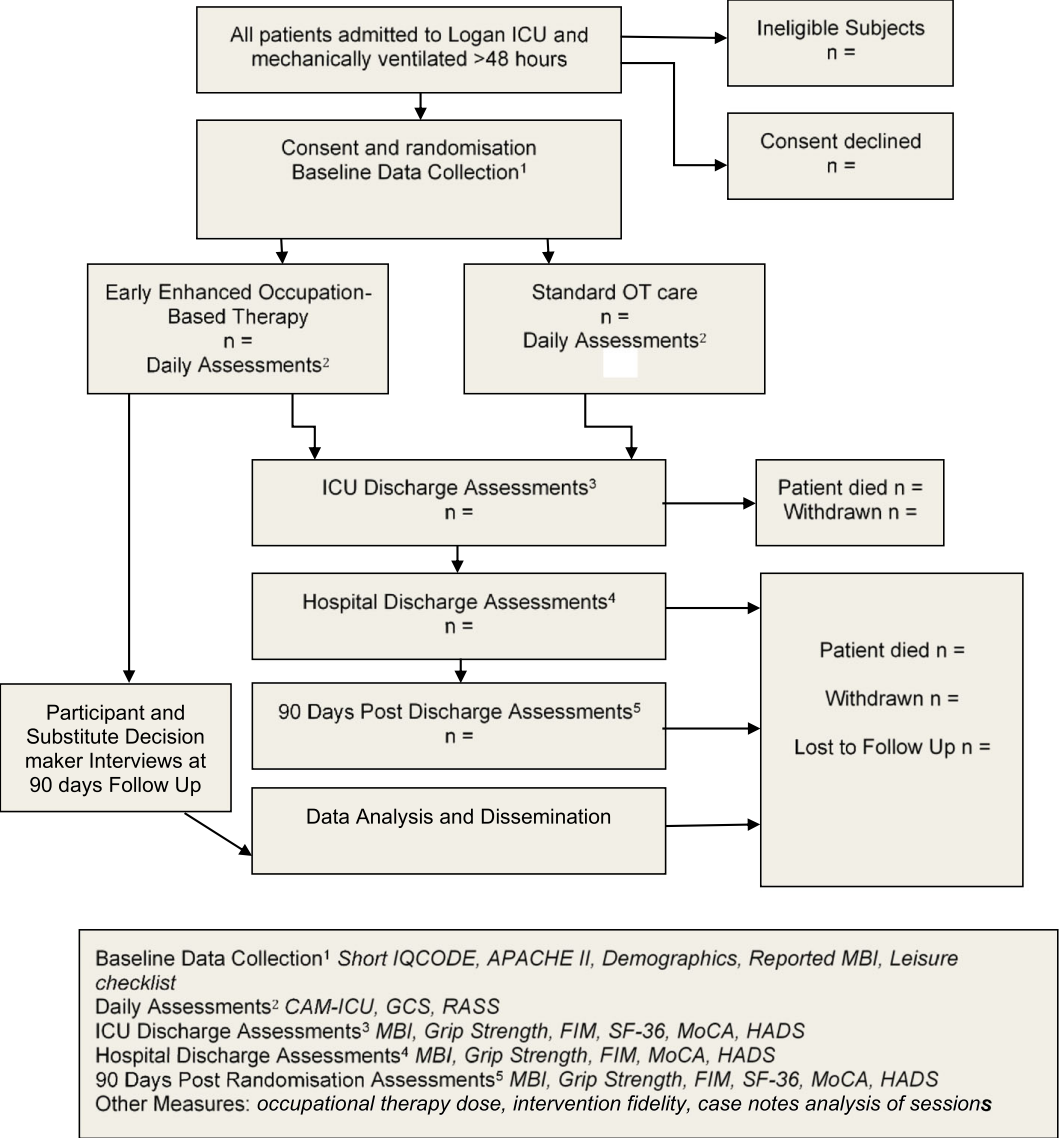
Trial flow chart for EFFORT-ICU

The trial, termed EFFORT-ICU, is a phase II feasibility trial incorporating a mixed methods design. The study follows the guidelines for feasibility trials outlined by the SPIRIT 2013 Statement [39]. EFFORT-ICU is a prospective single-centre, single blinded, equally randomised controlled trial (1:1) comparing standard occupational therapy care to an early enhanced occupation-based therapy. A study overview is shown in Fig. 1. The trial is registered as follows: Australia New Zealand Clinical Trials Registry (ANZCTR): ACTRN12618000374268; prospectively registered on 13 March 2018/https://www.anzctr.org.au.

### Ethics/governance

Ethical approval for the study from the Metro South Hospital and Health Service Human Research Ethics Committee (EC00167) and The University of Queensland was gained on 27 April 2018 for a 3-year recruitment study cycle. The trial may be independently audited by the ethics committees at any time. Any modifications to the protocol (for example changes to eligibility criteria, outcomes) will be approved by the ethics committees and communicated to all investigators and the trial registry.

### Reporting

The study results will be reported using the “CONSORT 2010 statement: extension to randomised pilot and feasibility trials” [40].

### Progression criteria to decide whether to proceed with RCT

As randomised controlled trials involve a significant organisational and financial input, the decision to progress from a pilot study requires clear and objective measures to ensure that staffing and patients benefit from participation in a study. The use of the guidelines and key information to consider, as proposed by Avery et al. (2017) [41], will influence the evaluation as to whether the trial should progress. The criteria for progression have been identified while considering the usual trial length for a grant funded study (2–3 years), with a projected trial recruitment of 100–150 patients to ensure improvements in clinical significance is achieved [24]. Targets for retention (75%) outcome measure administration (80%) and intervention completion (50%) were identified to minimise impact of missing data while aligning with clinical expectations for this cohort of patients (Table 1).

**Table 1:** Progression criteria for RCT

Domain	Proceed with RCT	Proceed with changes to protocol	Do not proceed unless problem resolution
Recruitment	Recruitment of 30 eligible patients within 6 months	Recruitment of eligible patients within 6–12 months	Unable to recruit 30 patients in 12 months
Retention	Minimum of 75% retention to follow-up	Minimum of 50 % retention to follow-up	Less than 50% retention to follow-up
Completion of intervention	At least 50% complete intervention for 40–60 min per day	At least 50% complete intervention for 20–40 min per day	Less than 50% complete intervention for 20 min per day
Outcome measures acceptability	Able to administer 80% of the outcome measures at each time point	Able to administer 80% of the outcome measures at each time point	Able to administer 80% of the outcome measures at each time point
Adverse events	No serious therapy-related adverse events during trial	Less than 5 serious therapy-related adverse events during trial	Five or more serious therapy-related adverse events during trial

### Participants

The study will be conducted in a Queensland level two adult eight-bed intensive care unit at Logan Hospital, Brisbane, Australia. All patients admitted to the intensive care unit will be included if they are aged over 18 years and are expected to require invasive mechanical ventilation for greater than 48 h.

Patients will be excluded from the trial if:

1. They are admitted to the intensive care unit but do not require mechanical ventilation.
2. They have been readmitted to the intensive care unit from the current hospitalisation.
3. A withdrawal of treatment is expected to occur within the next 24 h or they are not expected to survive the current intensive care unit admission.
4. They have a poor level of functional ability prior to admission (requiring carer assistance/high dependency level in activities of daily living as measured by a Modified Barthel Index score < 40).
5. They have a pre-existing severe cognitive deficit (as identified by completion of the short form IQCODE with an average score above 3.31–3.38).
6. They have a pre-existing significant mental health disorder impacting on participation.
7. They live interstate and would be unable to attend the 90 days post-randomisation follow-up in person.
8. They are unable to communicate in English.

Patients admitted over the weekend where occupational therapy intervention cannot be immediately initiated will be eligible to participate in the trial, and therapy will begin on the first working day.

The 90-day post-randomisation evaluations’ timepoint for the follow-up review date has been chosen based on the funding cycle. As this is a pilot feasibility study and the study is limited in duration, this time point was chosen to be sufficiently representative to identify if patients had ongoing concerns, and to ensure their memory of the therapy provision was current for the semi-structured interviews.

### Recruitment

A consecutive sampling model will be used where all patients who meet the eligibility criteria will be invited to participate. All patients admitted to the intensive care unit will be screened for eligibility on a daily basis by clinical staff. Patients meeting the study criteria will be referred to the research team within 24 h of admission. Written informed consent will be sought. Eligible participants will begin treatment after 48 h and before 72 h have elapsed since admission. The trial staffing has been adjusted to guarantee occupational therapy cover can be provided consistently Monday to Friday irrelevant of public holidays. This ensures that all patients have fair access to daily therapy within a working week irrespective of the time of year recruited.

### Consent

Participants will be invited to provide consent at the time of recruitment if able to do so. If due to medical conditions, the participant is unable to provide informed consent, the person responsible will be approached to provide consent on their behalf. In addition, informed consent will be sought from the person responsible to act as a significant other participant during the qualitative interviews in part 2. Once a participant is able to provide their own consent, they will be given an opportunity to continue with the trial or withdraw. Eligible participants will be informed that participation in the study is voluntary and if they choose not to participate, or if they choose to withdraw from the study after consenting, this will in no way affect their normal treatment at the hospital.

The study will be explained verbally by the chief investigator or nominated delegate. The participant or person responsible will be given the opportunity to read the information sheet and ask any questions prior to participation in the study. The participant or person responsible will be provided with a copy of the signed consent form and the information sheet and any other documentation discussed throughout the consent process.

### Randomisation and blinding

Following consent, participants will be randomly allocated to either the intervention group who will receive early occupation-based activity or the control group who will receive standard occupational therapy, medical, nursing and physiotherapy care. Patients will be randomised into the intervention and control groups via computer-generated numbers (http://www.randomization.com/) which will be sealed in consecutively numbered opaque envelopes. The production of the envelopes will be performed by research support staff who will not be involved in the interventions.

### Blinding

It will not be possible to blind the research team or the participants to group assignment. However, participant outcome measurement assessors will be blinded to participant assignment. The blind assessors will be other occupational therapists who work at the hospital but not in the intensive care unit, or with patients who have been transferred from intensive care.

### Interventions

#### Control group

The control group will receive usual occupational therapy care. At Logan Hospital, an occupational therapist attends daily clinical ward rounds within the intensive care unit. Patients are identified based on clinical needs and daily prioritisation guidelines for operation within an acute hospital. Core non-functional risk remediation activities such as splinting (for example to prevent foot drop) and pressure cushion provision are provided on an immediate basis when clinical need is identified. Functional therapy is completed at the discretion of therapists and clinical caseload weighting and may be absent in most units. The usual care group in this trial will therefore not receive any therapy unless an urgent splinting need is required to maintain range of movement. Administration of the daily outcome measures which includes the Multiple Status Questionnaire (MSQ) by the blinded bedside nurse is not anticipated to take longer than 10 min per day. Any intervention provided to the control group will be documented and included within the analysis.

#### Intervention group

Early enhanced occupation-based therapy is limited in the ICU. Identification of appropriate occupation-based therapy is dependent on cognitive (arousal, attention, executive functioning), physical (strength, co-ordination) and physiological restrictions (ventilator, cardiovascular status, medications). Co-morbidities, such as delirium, are assessed daily prior to the session and may result in session adaptation to maximise participation. Prior to engaging in early enhanced occupation-based therapy with the therapists, all patients will have received daily medical clearance by the treating physician. Clear physiological norms are established prior to participation to ensure that the occupation-based therapy is within the physiological capabilities of the participant [42]. A precautions checklist will be completed prior to each therapy session to ensure medical stability and clearance for activity participation. The checklist was created based on expert consensus guidelines for mobilisation in the intensive care unit and adapted to functional and cognitive participation requirements [42]. See Table 2 for contraindications to treatment. Monitoring of cardiovascular and respiratory status during early enhanced occupation-based therapy is completed by the bedside ICU nurse. Session termination will be under the guidance of the bedside nurse in collaboration with the occupational therapist and will be based on changes in cardiac and respiratory function as well as patient request to stop. Any immediate change that results in a contraindication being identified will lead to session termination. Further guidance on therapy discontinuation will be in line with published guidelines for exercise termination [43, 44] and includes a drop in systolic blood pressure, signs of myocardial infarction, unusual shortness of breath, arrhythmias, angina or signs of poor perfusion.

**Table 2:** Checklist for contraindications to intervention

- O_2_ Saturation < 90% - Respiratory Rate > 30 bpm - Fi O_2_ > 0.6 - PEEP > 10 cm H_2_O/ventilator dysynchrony - Any rescue therapies (NO, Prostatcyclin, prone) - Significant antihypertensive medications - MAP (below target, various support measures) - Bradycardia on significant pharmacological support - Tachycardia > 150 bpm - RASS > 2+ - Large open wound/uncontrolled active bleeding	

Patients will undergo sedation breaks during which targeted early enhanced occupation-based therapy will be delivered. The therapist will carry out the intervention to a maximum of 60 min per day, based on graded performance and localised adaptation from response level, such that sessions may be carried out twice a day (30 min each) to 3 times a day (20 min each) or a single session with a higher level patient (60 min). The measure of 60 min has been chosen to comply with perceived expectations of rehabilitation unit therapy intensity and duration per profession [45]. Where the full quota of therapy is unable to be provided, clear reasons for therapy termination will be gathered on the data form and analysed.

Prior to engaging the patient in early enhanced occupation-based therapy, participants will undergo simple cognitive and physical assessments to enable the therapist to adapt the chosen task to optimise performance and ensure that cognitive stimulation is appropriate. Pre-intervention measures include the following:

Richmond Agitation and Sedation Scale (RASS) [46] measures level of sedation and agitation. Patients will receive treatment if they fall within − 2 to + 2 scales. Patients falling on either extremes are deemed unsuitable for engagement at the current time point, although will be assessed regularly throughout the day prior to therapy, to ensure all opportunities for engagement are taken.

Glasgow Coma Scale (GCS) [47] measures arousal and response level in patients with severe injuries and is used to identify initial emergence of recovery post-sedation withdrawal. The highest score is 15 (fully oriented and alert) and the lowest score is 3 whereby a patient is considered unresponsive. The GCS will be completed prior to the treatment intervention as this will guide the therapist in providing either sensory stimulation [48, 49] or early enhanced occupation-based therapy.

The Confusion Assessment Measure ICU (CAM-ICU) [50] is an assessment of confusion in the early identification of delirium of patients within the intensive care unit. The presence of delirium will not limit participation within an early enhanced occupation-based therapy session. Strategies will be used to increase attention and encourage sequencing of short subtasks in order to complete a full grooming or self-care task.

The Short Portable Mental Status Questionnaire (MSQ) is a brief orientation checklist used to demonstrate current and previous memory linked to orientation. It is a 10-statement questionnaire that is easily administered at the bedside [51] and gives a therapeutic indication of ability to follow questions and commands and engage in therapy. A lack of orientation as evidenced through poor scoring on the MSQ will lead to the therapists including orientation cognitive tasks to facilitate cognitive recovery within the early enhanced occupation-based therapy. Strategies such as auditory retrieval priming (for example if yesterday was Tuesday then today is…) and errorless learning will be used to facilitate cognitive orientation recovery [52].

Early enhanced occupation-based therapy covers a range of activities of daily living including, but not limited to, grooming activities carried out in bed, grooming activities at the beside, grooming in the ICU bathroom, strip washing and self-care in the bed or bedside and showering in the ICU bathroom. Additional activities include leisure tasks related to board games, creative artworks, computer or iPad usage, adult mindfulness colouring in and functional grooming (manicure/makeup) and are identified based on completion of a leisure interest checklist as documented by the relevant next-of-kin or close family member (under consent guidelines). Activities are adapted through use of equipment, set-up, positioning and number of subtasks planned, to enhance participation outcomes and rehabilitation goals.

Cognitive stimulation tasks are often incorporated within the early enhanced occupation-based therapy through active engagement such as reality orientation, choice making and decision making, task sequencing directions, use of familiar objects, faces or smells, use of familiar music, participation in appropriately cognitively challenging games such as Sudoku and mindfulness colouring in, and reminiscence and recall challenges. The development of rapport supports engagement and enables the therapist to set appropriately challenging activities thereby enhancing participation and achievement of goals. Family engagement is another source of cognitive and functional activity and patients may ‘make something’ or video record a message for their family member where possible. Cognitive retraining involves the careful selection of appropriate strategies including but not limited to errorless learning, backward and forward chaining, vanishing cues and auditory priming [52].

Sensory stimulation tasks may be carried out in patients with a lower arousal level [48, 49, 53, 54]. Environmental adaptations (temperature of water, roughness of flannel) will be used to participate and adapt the tasks for heightened controlled sensory stimulation. Patients with lower levels of arousal will predominantly participate in regular shorter sessions with a focus on a tactile, auditory and visual stimulation within an occupation-based (hand-over-hand) task.

A manualised intervention strategy will be followed to ensure consistency of approaches; however, interventions will be personalised to facilitate increased participation and enhanced triggering of memories. A non-standardised leisure checklist or interview will be used to identify pre-morbid interests and lifestyle choices that can be incorporated within the session to enable appropriate choice of activity and topic discussion where relevant to stimulate cognitive recovery. The lifestyle/leisure checklist is based on the Person-Environment-Occupational Performance Model (PEOP) which serves as a framework for occupational therapy practice [55]. The checklist will also incorporate other regularly used client-centred checklists and models such as the Sunflower Chart developed by the Dementia Management and Advisory Service (DBMAS) for patients with a cognitive impairment, Model of Human Occupation (MOHO) [56] Interest Checklist and the culturally relevant Kawa Model [57]. This combination of approaches enables the therapist to develop a comprehensive list of activities and interests that could be used to facilitate participation and patient engagement. Table 3 lists the early enhanced occupation-based therapy and cognitive stimulation tasks carried out as part of the intervention.

**Table 3:** List of intervention therapy provision

Outcome measure				Occupation-based task	Cognitive stimulation task
GCS	RASS	MSQ	CAM	Function	Cognition
**3–8**	**− 2 to 0**	**0**	**+ve or −ve**	• Hand over hand facilitation of o Wiping face o Brushing teeth o Skincare o Oral care o Brushing hair o Washing upper body o Moisturising upper body	• Orientation prompts • Sensory stimulation—visual and auditory search and locate games • Communication/yes /no to simple biographical/physiological details—use adapted communication methods (visual, gestural based on ventilation status)
**8–12**	**− 1 to +2**	**0**	**+ve or −ve**	• Hand over hand facilitation of o Wiping face o Brushing teeth o Skincare o Oral care o Brushing hair o Washing upper body o Moisturising upper body • iPad activities including facetime relatives • Active assisted facilitation of o Wiping face o Brushing teeth o Skincare o Oral care o Brushing hair o Washing upper body o Moisturising upper body o Dressing while seated o Table top activities including games, creative work, cognitive stimulation, iPad challenge games o Upper limb activity programmes (e.g. theraputty, colouring in, creative work)	• Orientation • Sensory stimulation—visual and auditory search and locate games • Communication/yes/no • Reminiscence/recall therapy • Goal planning/structured day planning (participation and idea generation)
**13–14**	**0 to +2**	**1–10**	**+ve or −ve**	• Hoist transfer to commode for assisted showering or grooming at basin in ICU bathroom • Standing transfer to commode for assisted showering or grooming at basin in ICU bathroom • Stand transfer onto shower bench for assisted or supervised showering with task set-up depending on physical and cognitive abilities	• Orientation • Sensory stimulation—visual and auditory search and locate games • Communication/yes/no • Reminiscence/recall therapy • Goal planning/structured day planning (participation and idea generation)
**15**	**0 to +2**	**1–10**	**+ve or −ve**	As above but increasing choice making, initiation and independence required through task set-up, environmental challenges and attention / cognition challenges (rate of task performance, number of distractors, error recognition)	• Reminiscence/recall therapy • Goal planning/structured day planning (participation and idea generation) • Problem solving challenges, e.g. games, iPad use including facetime

### Adaptation of the MSQ and cognitive response tasks

Based on the frequency and nature of intensive care where patients may be ventilated or have poor attention span, adaptive strategies will be required to facilitate choice and communication in completing the MSQ and participating in cognitive tasks. This is managed via multiple methods:

1. Multiple choice written answers on laminated card: options vary between 3 and 10 per page pending arousal level. It is recommended that 3 options be used for ease with visual clues. Therapist can read out answers and request a yes/no pending communication, or if motor activity available, patient can select option.
2. Multiple choice verbal answers: maximum of 3 options read to patient with use of head nod or agreed communication method for yes/no (thumbs up/down, blink pattern).
3. White board or written answers to questions—provide adapted writing equipment (for poor grip strength) and whiteboard/paper positioned to allow maximum participation.
4. Mouthing or vebalisation in tracheostomy patients as able.
5. Alphabet board with large font for patient who can spell but not write—use technique as per communication method for eye scanning methods.

The intervention will be provided face-to face by the trained therapists and may occur in bed, at the beside or within the intensive care unit bathroom.

All occupational therapists expected to work within the intensive care unit will be trained and become familiar with the intervention manual prior to the study. All interventions will be delivered by the two trained specialist critical care occupational therapists based on the intervention manual. Training will be provided by the principal investigator (ARB) over a 3-month period incorporating observation and clinical reflection examples. Two reserve therapists whose primary caseload is not in the intensive care unit will be trained by ARB to ensure all functional sessions can be completed even in absence of usual critical care staff (for example due to annual leave or unexpected absence). Treatment fidelity will be monitored through case notes and discussion of therapy goals prior to session delivery. Any significant modifications (which exclude personalisation of therapy content delivery) will be discussed with the chief investigator prior to delivery.

#### Qualitative follow-up

Part 2 of this study includes a qualitative component and will be guided by interpretive description [58] to explore the lived experience of participants receiving the intervention and the perception of family members or the most involved significant other. At 90 days post-randomisation, all patients from the early enhanced occupation-based therapy group will be invited to participate in in-depth semi-structured interviews targeted towards exploring their experience of engaging within the early enhanced occupation-based therapy. In the event that the participant is too unwell to physically attend an interview, alternative arrangements, such as telephonic interview, will be offered.

The person responsible will be provided with a separate information and consent form to participate in the interview.

Interviews will be conducted by the principal researcher in a private, wheelchair accessible room at Logan Hospital. Funding for transport will be provided to facilitate attendance at interviews. Wheelchair accessible transport will be organised for participants who require specialist accessibility options. Interviews will be audiotaped and transcribed verbatim by a transcription service.

### Data collection

Data on recruitment, including number of eligible and consenting patients and reasons for non-consent, will be collected. Patient demographic data including age, gender, ICU admission diagnosis, co-morbid diseases, date of admission to ICU, ICU length of stay, hospital length of stay, hospital discharge destination and support level required on discharge from hospital will be collected from the medical records. Severity of illness will be measured using the Acute Physiology and Chronic Health Evaluation II (APACHE II) scoring system [59].

Duration of mechanical ventilation will be extracted from the Australia New Zealand Intensive Care Society ANZICS Adult Patient Database (APD). The time schedule for enrollments and administration of assessments and outcome measures is illustrated in Table 4. Retention data including number of withdrawals and reason for withdrawal will be collected. Data will be collected using multiple formal case report forms (CRFs) including pre-screening measures (inclusion/exclusion criteria on all screened patient admissions), admission demographics, pre-intervention checklist for decision making regarding therapy implementation, daily intervention pre- and post-measures, daily control group measures and collation of outcome measures at the three time points. The formal CRFs will be transcribed to an excel spreadsheet for analysis post trial completion. Data forms will be reviewed weekly by the primary investigator to ensure missing data occasions are minimised. Clinical case notes will be audited using a data collection audit checklist.

**Table 4:** Time schedule of enrolment and assessments for participants

Measure	Enrolment	Allocation to intervention	ICU Stay	ICU discharge	Hospital discharge	Follow up 90 days
Eligibility screen	x					
Informed consent	x					
Informant Questionnaire on Cognitive Decline in the Elderly (IQCODE)	x					
Demographic		x				
APACHE II		x				
Modified Barthel Index (MBI)	x (Reported)			x	x	x
Grip Strength (Dynamometer)				x	x	x
Functional Independence Measure (FIM)				x	x	x
Short Form (SF-36v2)				x		x
Hospital Anxiety and Depression Scale (HADS)				x	x	x
Confusion Assessment Measure (CAM-ICU)			Daily			
Glasgow Coma Scale (GCS)			Daily			
Short Portable Mental Status Questionnaire (MSQ)			Daily			
Richmond Agitation and Sedation Scale (RASS)			Daily			
Montreal Cognitive Assessment (MoCA)				x	x	x
Dose of Occupational Therapy			Daily			
Intervention fidelity checking through notes audit			Daily			

### Outcome measures

#### Primary outcome measure

Independence within activities of daily living will be measured using the Functional Independence Measure (FIM™). The FIM will quantify the participant’s functional and cognitive status at intensive care unit and hospital discharge, as well as at 90 days post-randomisation. This tool is validated for use in the critically ill population [60, 61] and provides reliable information regarding patient functional change during rehabilitation across various hospital and community settings [62]. The FIM will be conducted within 24 h of the expected discharge from the intensive care unit and hospital or on the Friday before the expected discharge, if likely to occur over the weekend. It will also be administered during the 90-day follow-up session. The FIM will be performed by an occupational therapist blinded to participant assignment groups and who does not actively work on the intensive care unit.

#### Secondary outcome measures

1. The Modified Barthel Index (MBI) [63] is used to measure functional ability and serves as a secondary measure included for its ability to detect change within self-care and functional tasks. The MBI is commonly used in hospital settings, although recent research has highlighted ceiling effects and lack of reliability [64]. The MBI also provides an additional source of comparison across multiple earlier critical care rehabilitation studies. The MBI will be used at multiple time points including allocation to intervention, intensive care unit discharge, hospital discharge and 90-day post-randomisation follow-up.
2. The Montreal Cognitive Assessment (MoCA) [65] is a screening measure of cognition and assesses multiple domains of cognition including attention, memory and visuospatial relations. It is commonly used within the acute and community setting and demonstrates high criterion and convergent validity within the acute care setting [66]. The MoCA will be administered at discharge from intensive care unit, hospital and 90-day post-randomisation follow-up using alternate versions at each time point [67].
3. Grip strength will be measured using a dynamometer (Jamar) [68] at discharge from intensive care unit and hospital and 90-day post-randomisation follow-up. Grip strength is used as a measure of intensive care unit-acquired weakness (ICUAW) and infers a relation between weakness and inability to complete functional activities independently [69].
4. The Short-Form (36) Health Survey (SF-36v2™) will provide a baseline and post discharge measure of participants’ health-related quality of life (HRQOL). The SF-36v2™ is a validated and reliable tool [70] and has been further validated within the critical care setting [71]. This will be completed at intensive care unit discharge and 90-day post-randomisation follow-up.
5. Sedation and delirium status will be measured using the validated Richmond Agitation Sedation Scale (RASS) [46] and the reliable and validated Confusion Assessment Method for ICU (CAM-ICU) [50]. These scores are regularly administered by nursing staff throughout a 24-h period. RASS and CAM-ICU scores immediately prior to and following early occupation-based interventions will be collected for additional analysis. RASS and CAM-ICU scores for the control group participants will be collected daily at 9am.
6. The Glasgow Coma Scale (GCS) [72] is used to evaluate the level of consciousness in patients with neurological conditions or severe injury [73] and will be collected daily at 9 am in the control group, and pre-treatment in the intervention group.
7. Hospital Anxiety and Depression Scale (HADS) [74] is a measure of emotional distress regarding anxiety and depression. It has been validated with the use of critical care survivors [75]. It will be administered at discharge from intensive care unit, hospital and 90-day post-randomisation follow-up.
8. The Informant Questionnaire on Cognitive Decline in the Elderly (IQCODE) (short form) [76] is a reliable and validated measure used to screen for cognitive dysfunction. It will be used as a screening tool for eligibility to the trial and will be completed by the participant’s person responsible.
9. The Short Portable Mental Status Questionnaire (MSQ) [51] is a brief orientation checklist used to demonstrate current and previous memory linked to orientation. It is a 10-statement questionnaire that is easily administered at the bedside. It will be completed pre-and post-treatment in the intervention group, and at 9 am in the control group.

### Data management

All data for the individual participants will be collected by the chief investigator and co-investigator therapists who will administer the interventions. Participants will be allocated a participant number for the trial. All case report form (CRF) data will be entered into a password protected data sheet for analysis and storage.

### Sample size

A sample size of 30 participants has been chosen, based on recommendations regarding pilot and feasibility trials evaluating outcomes [77]. Fifteen patients from the intervention group will be included within the qualitative component carried out at 90 days post-randomisation.

### Data analysis

All data from the case report forms (CRF) will be entered into a purposefully designed Excel database and exported into SPSS version 22.0 for analyses. Descriptive statistics will be used to determine the distributions of the data for the intervention and control groups and to test whether statistical assumptions for parametric tests are achieved. Imputation will be used to correct for missing data. Continuous variables that are normally distributed will be compared between the groups at each follow-up using an independent groups *t* test. Effect sizes will be calculated using Cohen’s *d*. Non-parametric analysis will be conducted for continuous variables that are not normally distributed. Categorical variables will be compared with the chi-square statistic. A *p* value of 0.05 will be considered statistically significant. Where appropriate, analyses will be reported with mean differences and 95% CI. Previous values from the same patient will be used for missing data.

In particular, objectives will be analysed as follows:

1. The utility of the selection of outcome measures will undergo sensitivity analyses in relation to the ease with which they can be administered and will relate to missing data and the identification of both univariate and multivariate outliers. The effect size for the primary outcome variable will be used to determine the required sample size for a fully powered trial.
2. Recruitment and consent rates will be collected and analysed to determine the feasibility of recruiting form the chosen population. The inclusion and exclusion data (screening log) will be summarised and will influence further protocol changes when considering progression to a full scale randomised controlled trial.
3. Retention rates in response to follow-up procedures (attendance at interviews) will be analysed to identify any trends that may influence further trial progression.
4. The intervention data will undergo sensitivity analyses in relation to non-compliance, the intention to treat per protocol in addition to as treated. Protocol violations regarding the intervention manual strategies will be noted.
5. The intervention effect will be analysed through the use of the above outcome measures, and further analysis of case notes will reveal common trends in treatment adaptation or selection, based on the structure of the intervention manual.

Patient experience, satisfaction with treatment and perceived benefit of participation within the rehabilitation group will be gathered through the semi-structured interviews. The interview data will be transcribed and analysed using thematic analysis [78] to identify key perceptions and self-reported experience and highlight factors that may contribute to future clinical care design. The data will be analysed inductively as little is known regarding the patient experience; therefore, deductive categories cannot be imposed on the data. Two members of the research team will independently code a portion of the data.

### Analysis of session content

All case notes of patients seen by occupational therapy will be audited using a pre-designed data collection form, analysed to categorise common therapeutic interventions used, and determine fidelity with respect to adherence to manualised intervention protocol. Deviations from the protocol will be reviewed. Case notes for patients experiencing usual care will also be analysed to identify if any cross-over of groups occurred. Number of interventions delivered will be recorded for each intervention participant, detailing reasons for missed therapy sessions.

### Adverse event management

There is no anticipated harm associated with participating in the early rehabilitation programme. All occupational therapists carrying out the interventions will be trained and experienced critical care staff. Unforeseeable changes in medical condition may influence the type and content of therapy sessions and there may be a period where therapy is reduced under the guidance of the medical team until organ system stabilisation has occurred. Therapy will be initiated as soon as both medical and allied health teams identify that safe mobilisation practices can be re-implemented. The nature of medical complications impacting on delivery of the intervention will be documented.

Occupational therapy staff completing the outcome measures are familiar with the complex caseload of patients and will be sensitive to the vulnerable nature of the questions and outcome measures used. Staff will invite participants to have a family member/support person present and will cease the assessment if a participant becomes distressed. Emotional and physical safety of the vulnerable participants will be their primary concern.

The qualitative interviews will be carried out by an experienced critical care occupational therapist who is familiar with the sensitive and vulnerable nature of the participants. It is acknowledged that asking participants to recall their time in intensive care has the potential to be distressing. Questioning will cease if the participant becomes distressed, and a family member/support person will be invited to attend should they not already be present. Participants will be provided with further information regarding community support services or organisations should they need ongoing support with coping.

### Confidentiality and security

All patient data (paper copy and secure computer files) pertaining to the study will be stored maintaining confidentiality in accordance with local legislation on privacy and the use of health data.

Confidentiality of all patient information will be safeguarded through coding mechanisms and stored in secure locked conditions with access limited to study personnel. Electronic files will be password protected. The chief investigator will maintain the confidentiality of all study documentation and participants and take measures to prevent accidental or premature destruction of these documents, and prevent access to this data by any unauthorised third party. Information will be de-identified. Each participant will be given a unique identifier linked to a master list. The master list will be stored separately to the data and will only be accessed by the chief investigator.

The investigator will retain study documents for at least 15 years after the completion of the study. Study documents will be disposed of securely after 15 years—paper documents will be shredded, and computerised data will be permanently erased and back-ups physically destroyed.

## Discussion

Early rehabilitation within critical care units has been shown to be effective in multiple studies [17, 24]; however, the occupational therapy approaches to enhance long-term recovery require further investigation especially in mechanically ventilated patients. This trial aims to explore the feasibility of providing occupational therapy to mechanically ventilated patients in a critical care setting. It aims to identify approaches that may be beneficial to patients and family members surviving a critical care stay, as well as defining where priorities in service provision may lie. The ability to optimise recovery with clearer multidisciplinary input may strengthen future collaboration to drive an early rehabilitation model. Analysis of day to day therapy provision, the ability to adapt and engage patients along a continuum of sedation and immobilisation variables will provide clarity about the types of interventions that are best completed in the critical care setting by an occupational therapist. While this study is not powered to detect clinically significant change, it will provide information that will enable the design of a phase III trial including sample size calculations. A dissemination plan has been developed that includes publication in peer-reviewed journals, webinars and conference presentations and posters.

## Limitations

While every effort has gone into designing a trial that is feasible to implement, there are inherent limitations including the exclusion of patients admitted to the unit which may reduce the generalisability of the therapy. Specifically, patients that would benefit from treatment but have mental health or other confounding factors such as distance from treatment hospital that may influence follow-up procedures and attendance are excluded. Further limitations include the lack of blinding of therapists delivering the therapy in the trial as this simply is not feasible given the funding constraints and a lack of suitably experienced occupational therapists at the trial site. Therefore, the same therapists providing intervention will also provide the usual care to the control group. Although there is a manualised intervention protocol, there is some personalisation of therapy required to increase participation. The intervention is occupation-focused and clients differ in their occupational roles; therefore, each client will require minor changes to individualise the treatment. Variations in content in intervention trials may be considered a limitation but also reflect actual clinical practice potentially enabling validated interventions to be more readily translated into practice. Other risks exist in the trial in relation to the rate at which clients can be recruited, the impact of exclusion criteria on recruitment rate, and attrition at the follow-up timepoint.

## Conclusions

Although still in its infancy regarding role establishment and clinical effectiveness within intensive care units [22], occupational therapists are key professionals who are able to assist the early rehabilitation philosophy in intensive care by addressing and challenging current self-care and cognitive processes, factors which are evident in those presenting with post-intensive care syndrome. This feasibility trial will provide an approach towards the implementation of functional occupational therapy intervention in the critical care setting to promote a consistent manner of introducing meaningful occupations that influence long-term recovery. It will add to the growing body of evidence supporting early and effective rehabilitation in intensive care units enabling a shift in outcomes from mortality to independence.

## Trial status

The trial is recruiting participants but is paused due to COVID-19.

## Supplementary Information

**Additional file 1: Appendix A.** Consent form. Appendix B: Participant and Substitute Decision Maker Interview Guide. Appendix C: TIDieR Checklist Indicating Sample Intervention for Upper Body Grooming

## Data Availability

The full protocol is available in the Australia New Zealand Clinical Trials Registry (ANZCTR) (ACTRN12618000374268). Participant-level data set and statistical code may be requested from the principal author following publication of the trial.

## Abbreviations

ICU: Intensive care unit
OT: Occupational therapist
PICS: Post intensive care syndrome
