# Supplementary material for: A comparison of standard occupational therapy versus early enhanced occupation-based therapy in a medical/surgical intensive care unit: study protocol for a single site feasibility trial (EFFORT-ICU)
Source: Pilot Feasibility Stud. 2021 Feb 18;7:51. doi: 10.1186/s40814-021-00795-2 (PMC7889705; doi:10.1186/s40814-021-00795-2)
Supplement: Supplementary file 1 — Additional file 1: Appendix A. Consent form. Appendix B: Participant and Substitute Decision Maker Interview Guide. Appendix C: TIDieR Checklist Indicating Sample Intervention for Upper Body Grooming [file 40814_2021_795_MOESM1_ESM.docx]

**APPENDIX A: CONSENT FORM**

**PARTICIPANT INFORMATION AND CONSENT FORM – PERSON RESPONSIBLE (ENROL)
Version 3.0, Dated 2^nd^ October, 2018.**

**Full Project Title:**

The effectiveness of early functional occupation-based retraining therapy in a medical / surgical intensive care unit: study protocol for a single-site feasibility trial (EFFORT-ICU)

**Principal Investigator:** Mrs Andrea Rapolthy-Beck

Logan Hospital

**Associate Investigators:** Professor Jennifer Fleming

Dr Merrill Turpin

University of Queensland

Dr Hayden White

Ms Kellie Sosnowski

Mrs Simone Dullaway

Logan Hospital

This Participant Information and Consent Form is nine (9) pages long. Please make sure you have all the pages.

**1. INTRODUCTION**

You are invited to allow your relative / friend, for whom you are responsible to take part in this research project. This Participant Information and Consent Form – Person Responsible (Enrol) contains detailed information about the research project. Its purpose is to explain to you as openly and clearly as possible the purpose and procedures involved in the project. Before agreeing for your relative / friend to participate, it is important for you to read and understand the information in this document.

The study is being conducted within this institution by Mrs Andrea Rapolthy-Beck.

Please read this information carefully. Please ask the principal investigator Mrs Andrea Rapolthy-Beck or one of the study team members to explain any words in this document that you do not understand. Also ensure all your questions have been answered to your satisfaction before signing this consent form. Before deciding whether or not for your relative / friend to take part, you might want to talk about it with another relative or friend.

Participation in this research project is voluntary. If you do not wish your relative / friend to take part, you do not have to. Your decision whether to allow your relative / friend to take part or not to take part will not affect your relative / friend’s relationship with the Logan Hospital or treating team and care.

When your relative / friend regains capacity a member of the research team will provide the same information and discuss the research project with your relative / friend. Your relative / friend will then be given the opportunity to continue or withdraw consent.

If you decide you want your relative / friend to take part in the research project, you will be asked to sign the consent section. By signing it you are telling us that you:

- Understand what you have read
- Consent for your relative / friend to take part in the research project
- Consent for your relative / friend to the use of their personal and health information as described

You will be given a copy of this participant information and consent form to keep.

**2. PURPOSE OF THE STUDY**

The aim of this research project is to determine if a new treatment approach improves the physical skills (e.g. strength) and selfcare skills (e.g. being able to wash their face or make a choice) of patients on ventilation in intensive care. The new treatment approach uses early stimulation of thinking skills (e.g. concentration and memory) as well as help to carry out simple daily tasks delivered by an occupational therapist.

**3. STUDY PROCEDURES**

The study will be carried out at the Logan Hospital Intensive Care Unit (ICU). This study involves taking part in early rehabilitation focusing on the recovery of thinking (cognitive) and physical (strength) skills within activities that were completed on a daily basis prior to your relative / friend’s admission, for example washing their face or body, brushing their teeth, or concentrating in order to be able to join in therapy.

If you choose for your relative / friend to be involved in this study and they meet all eligibility requirements, he or she will be randomly allocated to a treatment group that receives usual occupational therapy care or a group that will receive the new treatment approach.

Eligibility for the study will be determined by an initial screening process. In particular we will ask you if your relative / friend had:

1. Difficulty with thinking / memory before being admitted to ICU
2. Dependence in their usual self-care activities (e.g. requiring a carer to help with showering or dressing)

Your relative / friend may be seen daily by an occupational therapist and will take part in activities according to how awake or tired they are. The occupational therapist will ask you about your relative / friend’s interests and abilities in order to design a treatment plan that will be interesting and individual to him or her. This helps get a better response during treatment sessions.

Three months after the trial started, you and your relative / friend will be asked to return to the hospital briefly for a follow up visit with the occupational therapist, to complete some tests that will show how well your relative / friend has recovered. You and your relative / friend may also be asked to join in an interview to explore how you both felt about being involved in the early rehabilitation. We are aware that this may bring up sensitive issues for you and your relative / friend, and you may not wish to visit your experience. You will be supported throughout the interview by an experienced occupational therapist and provided with additional information regarding community supports if needed. If your relative / friend does not feel well enough to attend the interview, we can discuss closer to the time to find a suitable date and venue.

The initial treatment will take place in the Intensive Care Unit. The follow up visits will be held in private rooms located at Logan Hospital. You will receive full details regarding room location via telephone prior to your interview date.

**4. POSSIBLE BENEFITS AND RISKS**

There may be no benefits to your relative / friend’s participation, however there is no anticipated harm linked with taking part in the early rehabilitation which will be carefully conducted. Your relative / friend’s participation will help researchers better understand how early self-care activities can improve the future skills of patients in intensive care.

If eligible for the study, there are no known or foreseeable risks or side effects associated with your relative / friend’s participation. Unforeseeable changes in medical condition will influence the type and content of therapy sessions and your relative / friend may experience a period where therapy is reduced under the guidance of the medical team to allow their body to recover. However, therapy will always be re-started as soon as both medical and allied health teams feel it would be safe and beneficial.

If during the interview you or your relative / friend feel distressed, we will stop the interview and give you information regarding community support services or organisations that can help you.

If at any time you feel there is a problem related to your relative / friend’s involvement in this study, you should immediately alert the staff caring for your relative / friend.

**5. ALTERNATIVES TO PARTICIPATION**

Participation in any research project is voluntary. You are free to withdraw your relative / friend from the research project at any stage if for any reason you choose. Your decision whether to allow your relative / friend to take part, or not to take part and then withdraw will not affect you or your relative / friend’s relationship with the Logan Hospital or your relative / friend’s treating team. If you decide to withdraw your relative / friend from this project, please notify the principle investigator or one of the study team members so that procedures for withdrawal can be completed.

**6. COMPENSATION FOR INJURY OR COMPLICATION**

If your relative / friend suffers any injury or complication as a result of this research project, you should contact the principal investigator / study team as soon as possible, who will arrange appropriate medical treatment. If your relative / friend is eligible for Medicare, your relative / friend can receive any medical treatment required to treat the injury or complication, free of charge, as a public patient in any Australian hospital.

**7. COSTS**

There are no costs associated with participating in this research project, nor will you / your relative be paid. There is limited funding available to support those with financial hardships in attending follow up visits and interviews.

**8. PRIVACY, CONFIDENTIALITY AND DISCLOSURE OF INFORMATION**

Any information obtained for the purpose of this research project that can identify your relative / friend will be treated as confidential and securely stored by the study team. It will be disclosed only with you or your relative / friend’s permission, or as permitted by law. The results of this research project may be submitted to regulatory authorities, published, or presented at scientific meetings and may be used for further research, but your relative / friend’s data will be anonymous and will not be identifiable.

**19. FURTHER INFORMATION AND WHO TO CONTACT**

The person you may need to contact will depend on the nature of your query.

When you have read this information, Mrs Andrea Rapolthy-Beck, the Principal Investigator or one of the study team members will discuss it with you further and answer any questions you may have.

If you want any further information concerning this project, you can contact the principal investigator, Mrs Andrea Rapolthy-Beck, via telephone (07) 3299 8858.

All research in Australia involving humans is reviewed by an independent group of people called a Human Research Ethics Committee (HREC). The ethical aspects of this research project have been approved by the Metro South Hospital and Health Service Human Research Ethics Committee (EC00167).

This project will be carried out according to the National Statement on Ethical Conduct in Human Research (2007). This statement has been developed to protect the interests of people who agree to participate in human research studies.

If you have any ethical issues or concerns about the conduct of this study or any questions about being a research participant in general, you can contact the HREC Coordinator, Metro South Hospital and Health Service, Human Research Ethics Committee on (07) 3443 8049.

**Thank you for taking the time to consider this study.** A copy of this information sheet and signed consent form will be given to you to keep.

**
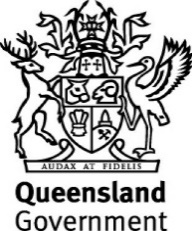
**

**PARTICIPANT INFORMATION AND CONSENT FORM – PERSON RESPONSIBLE (ENROL)
Version 3.0, Dated 2^nd^ October, 2018.**

**Full Project Title:**

The effectiveness of early functional occupation-based retraining therapy in a medical / surgical intensive care unit: study protocol for a single-site feasibility trial (EFFORT-ICU)

**Principal Investigator:** Mrs Andrea Rapolthy-Beck

Logan Hospital

**Associate Investigators:** Professor Jennifer Fleming

Dr Merrill Turpin

University of Queensland

Dr Hayden White

Ms Kellie Sosnowski

Mrs Simone Dullaway

Logan Hospital

Declaration by Participant

- I have read and understand the Participant Information Sheet or someone has read it to me in a language that I understand on the above named research project and have discussed the study with ____________________________ (investigator/ delegate).
- I have been made aware of and understand the purposes, procedures and risks of the research described in the project as far as they are known by the researchers.
- I understand that participation of my relative / friend in this study will allow the researchers to have access to my relative / friend’s medical record, and I agree to this.
- I also understand that any personal information will remain confidential.
- I have had opportunity to ask questions and I am satisfied with the answers I have received.
- I freely agree for my relative / friend to participate in this research project as described and understand that I am free to withdraw my relative / friend at any time during the study without affecting my relative / friend’s health care.
- I hereby agree for my relative / friend to participate in this research project.

**Name of Participant** (please print)

___________________________________________________________________

**Name of Person Responsible** (please print)

___________________________________________________________________

**Relationship to Participant** ____________________________________________

**Signature**__________________________ **Date /Time**________________________

**Name of Study Investigator / Delegate** (please print) ___________________________________________________________________

**Signature**__________________________ **Date /Time**________________________

^†^ A member of the research team must provide the explanation of, and information concerning, the research project.

**
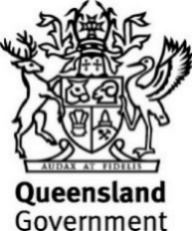
**

**REVOCATION OF CONSENT FORM – PERSON RESPONSIBLE
Version 3.0, Dated 2^nd^ October, 2018.**

**Full Project Title:**

The effectiveness of early functional occupation-based retraining therapy in a medical / surgical intensive care unit: study protocol for a single-site feasibility trial (EFFORT-ICU)

**Principal Investigator:** Mrs Andrea Rapolthy-Beck

Logan Hospital

**Associate Investigators:** Professor Jennifer Fleming

Dr Merrill Turpin

University of Queensland

Dr Hayden White

Ms Kellie Sosnowski

Mrs Simone Dullaway

Logan Hospital

I hereby wish to WITHDRAW my relative / friend from the study described above and understand that such withdrawal WILL NOT jeopardise any treatment or my relationship with the Logan Hospital for my relative / friend.

I agree to the use of my relative / friend’s data that was collected prior to my withdrawal of consent. **Yes / No** (please circle)

**Name of Participant** (please print)

___________________________________________________________________

**Name of Person Responsible** (please print)

___________________________________________________________________

**Relationship to Participant** ____________________________________________

**Signature**__________________________ **Date /Time**________________________

**Appendix B: Participant and Substitute Decision Maker Interview Guide**

*Introduction:* Thank you for taking the time to meet with me to today. During this interview, I am hoping to explore how you felt and what you think about your recent stay in the intensive care unit. In particular I want to focus on the occupational therapy (or OT) sessions you had with myself or the other therapist. If you are not sure what they were exactly, that’s ok, as I will try to give some suggestions of what we may have done with you. Sometimes it can all be a blur!

There is no right or wrong answer as this interview is designed to explore your experience. With your permission, I’m going to be recording this interview so that I can go over what you said and it helps me to remember our conversation. We have an hour together to talk about your experience so there is no rush. If you want to stop at any time, you just need to let me know and we can take a break.

1. Can you tell me in your own words why you were recently admitted to the intensive care unit at Logan Hospital?
2. Can you tell me how much you remember about your stay in ICU?
3. Do you recall working with an occupational therapist (myself, Andrea, or Simone) while you were in the intensive care unit?
4. Can you tell me a little about what you may have done with us during your treatment sessions?
5. How did these sessions make you feel?
6. What did you like about the sessions, if anything? Can you describe any benefits to you of having occupational therapy while you were in ICU?
7. Was there anything you didn’t like about the OT sessions? Do you have any recommendations for what the OTs could do differently with patients in the future?
8. Overall what was your experience of working with occupational therapists in the intensive care unit?
9. Can you tell me a bit about your time after ICU, while you were on the ward?
   1. What health professions did you see?
   2. What was your daily routine like?
10. Can you tell me about your time at home?
    1. What was your routine like? What did you do?
    2. What did you find easy? What did you find difficult?
    3. Are you experiencing any ongoing concerns?

**APPENDIX C: TIDieR Checklist Indicating Sample Intervention for Upper Body Grooming**

| **Name** | Provision of enhanced occupation-based therapy in a surgical/medical intensive care unit | | |
| --- | --- | --- | --- |
| **Rationale (Why)** | Goals: 1) Encourage cognitive attention and participation in session for 30 minutes | 1. Facilitate reach and grasp (motor strnbth in functional self-care grooming while in bed) |  |
| **What** | 1. Cognitive: Orientation prompting, location of grooming items around bedside room | 1. Physical; Reach and grasp for: a) washing face, drying face, upper body washing while seated upright in bed. Ability to reach forwards with min physical support |  |
| **Who provided** | Therapist provided; trained ICU occupational therapist (name) |  |  |
| **How** | Face to face delivery, assistance with environmental set-up (items within reach). Use of communication board or method as agreed from clinical notes | | |
| **Where** | ICU Environment: high sitting within bed, use of bedside table | | |
| **When and How much** | Session start: 10:00, session length: 30 minutes  Session closure; 10;30 | Casenotes charted to identify participation aspects, number of prompts, ability to recognise familiar objects, ability to sequence task in correct manner. Amount of physical assistance required to use objects appropriately |  |
| **Tailoring** | Equipment: e.g. light weight flannel, built up toothbrush  Timing: numre of prompts  Amount of set-up for task (all objects placed on trable or single item based on attention ability) | | |
| **Modifications** | Length of session, number of tasks completed (full upper body grooming vs face wash only) | | |
| **How well** | Planned: number of self-care tasks completed. | Refer to completed case notes for session outcome |  |
